# Improving accuracy of age estimates for insect evidence—calibration of physiological age at emergence (*k*) using insect size but without “*k versus* size” model

**DOI:** 10.1093/fsr/owad049

**Published:** 2024-02-25

**Authors:** Jędrzej Wydra, Łukasz Smaga, Szymon Matuszewski

**Affiliations:** Laboratory of Criminalistics, Adam Mickiewicz University, Św. Marcin 90, Poznań 61-809, Poland; Wielkopolska Centre for Advanced Technologies, Adam Mickiewicz University, Uniwersytetu Poznańskiego 10, Poznań 61-614, Poland; Department of Mathematical Statistics and Data Analysis, Adam Mickiewicz University, Uniwersytetu Poznańskiego 4, Poznań 61-614, Poland; Laboratory of Criminalistics, Adam Mickiewicz University, Św. Marcin 90, Poznań 61-809, Poland; Wielkopolska Centre for Advanced Technologies, Adam Mickiewicz University, Uniwersytetu Poznańskiego 10, Poznań 61-614, Poland

**Keywords:** forensic entomology, postmortem interval, insect age, accuracy, data analysis

## Abstract

Postmortem interval may be estimated based on the age of insect evidence collected on a death scene. Reference data that are used in such estimation frequently comprise thermal summation constant (i.e. *k*), which is equal to the insect age upon completion of immature development expressed in accumulated degree-days or degree-hours (ADD or ADH). Essentially, *k* is a central point of an insect group and it may poorly represent insect evidence that is near the limits of variation for the group. Accordingly, it was postulated to calibrate *k* for particular insect evidence and insect size and sex were found to be useful for this purpose in some of the species. However, the calibration is only possible by using the model that correlates *k* with insect size. Since very few such models were published, this lack of data limits the calibration of *k* in forensic casework. In this article, we develop a formula that is useful for the calibration of *k* without the use of “*k* versus size” model (and related datasets). The formula uses *k* from the general thermal summation model for a species (with its standard error), size range for the species (retrieved from entomology literature), and size measurements for particular insect evidence. The calibration of *k* with the formula was validated using the *Creophilus maxillosus* (Coleoptera: Staphylinidae) and *Necrodes littoralis* (Coleoptera: Silphidae) datasets. It was particularly useful while analyzing unusually small and large insects, in case of which the formula reduced the inaccuracy of *k* from the general model on average by ~25 ADD in *C. maxillosus* and ~40 ADD in *N. littoralis*. We discuss the limitations and prospects of the calibration protocol that employs the formula.

## Introduction

Insects collected on human cadavers are frequently used as evidence to estimate the postmortem interval (PMI) [1]. Usually, forensic entomologists base these calculations on the age of the most developmentally advanced immature insects; in a typical scenario age of such evidence delineates the minimum PMI [2]. Insect age may be expressed using physiological time, i.e. the combination of time and temperature in the form of thermal accumulation units, e.g. degree-hours or degree-days [3]. Physiological age upon completion of immature development (i.e. age at emergence or age at maturity) may be used to estimate PMI_min_ [4]. It is necessary, in particular, for the estimation of PMI_min_ based on the empty fly puparia or teneral insects [5]. This age is referred to as the thermal summation constant or *k* [6, 7]. When live insects are collected at a death scene, they are frequently reared until the emergence of adults. In such cases, *k* may be used to calculate the physiological age at the moment of collection by subtracting degree-days accumulated during rearing in the laboratory from *k* [4].

Although thermal summation constants are sometimes regarded as truly constant for an insect species or population, plenty of data have been accumulated that demonstrate their substantial variation. For example, *k* was found to differ between the sexes within a species [8–10], between different geographic populations of a species [11–13] and between different sized insects within the local population of a species [14, 15]. Essentially, *k* is a central point (an average) of an insect group (i.e. a species or its local population). As such, it poorly represents insects that are near the limits of variation for the group, especially when variation within the group is large. For these reasons, it was postulated that thermal summation values to be used in the estimation of insect age in forensic entomology should be calibrated for particular insect evidence [11, 14, 15]. Insect size was found to be particularly useful for such calibration, due to its significant relationship with *k*, as demonstrated in the case of forensically useful beetles: a rove beetle *Creophilus maxillosus* L. (Staphylinidae) and a carrion beetle *Necrodes littoralis* L. (Silphidae) [14, 15]. In effect, it has been suggested to measure the size (length or weight) of an insect at maturity and based on this measurement to estimate its true *k* [14]. When combined with insect sex, size measurements enabled to reduce the inaccuracy of *k* from the general thermal summation model on average by ~14% in the case of *C. maxillosus* and ~35% in the case of *N. littoralis* [14, 15].

In order to calibrate *k* using insect size (and sex), it is necessary to use the model that regresses *k* against size, at best separately for males and females of a species. Such models are derived based on the results of insect development studies, in which development and size data are collected for individual insects. Unfortunately, the necessity to use ‘*k*  *versus* size’ model for a species may limit the use of this calibration protocol in forensic casework. So far, such models were published only for *C. maxillosus* and *N. littoralis* [14, 15]. In this article, we develop and validate a formula for the calibration of *k,* without the use of “*k versus* size” model (and related datasets). The formula enables calibration based on thermal summation data for the species (*k* from the general thermal summation model and its standard error), size range for the species, which is usually available in entomology literature and size measurements of particular insect evidence.

## Materials and methods

The formula was derived based on Reduced Major Axis (RMA) regression. The RMA was chosen, since it is better than the ordinary Least Square Regression, when this is unclear which variable should be treated as the dependent one. We considered the relationship between the “true *k*” and insect length, because data on insect length are available for most insect species. However, major conclusions of this study are also applicable to insect weight and other measures of insect size.

Datasets on *C. maxillosus* (173 observations) and *N. littoralis* (954 observations) were used to validate the formula. The datasets originated from earlier studies supervised by the senior authors [14, 15]. They consist of lengths (measured with half-millimeter accuracy) and the “true *k*” (calculated for fixed temperatures and observed time of insect development) for individual insects. The data were collected during the laboratory experiments on insect development in forensic entomology (for detailed methods see [14, 15]).

For the purpose of validation, we performed two analyses using datasets for both species. In each species, we divided individual observations into length classes (insects with the same value of length were included in a class; 18 classes for *N. littoralis* and 19 for *C. maxillosus*) and calculated medians of the “true *k*” in each class. Such transformation was necessary because most insects were contained in classes close to the mean length. Extreme classes had significantly less observations. In effect, the model could be biased. Using medians from each class was a way to remove this bias. These datasets (consisting of medians only) were used in further analyses.

In the first analysis, we fitted RMA model to the true data (medians of the true *k* and insect lengths). Then, we calibrated *k* using the formula and the same insect lengths and used these calibrations to outline the ${K}_{\text c}$ model. We also outlined the “model” for constant *k*. Fit of these three models to the actual data was compared using the mean squared errors (MSE). While calibrating *k* we used minimal and maximal lengths taken from the relevant datasets: 15–24 mm for *C. maxillosus* [14] and 12–22 mm for *N. littoralis* [15].

The second analysis was made on unusually small and large insects only, to identify the true benefits of the calibration. The *C. maxillosus* sample consisted of the beetles <19 mm (~33rd percentile) and >21 mm (~66th percentile). The *N. littoralis* sample consisted of the beetles <16.5 mm (~33rd percentile) and >19.5 mm (~66th percentile). Using these samples we calculated the RMA, the ${K}_{\text c}$, and the constant *k* models. The significance of differences between the models in the squared errors was tested using ANOVA test and Least Significant Difference *post hoc* test (with Benjamini & Hochberg *P*-value adjustment [16]).

All the analyses were conducted with the R software (R version 3.6.3, 29 February 2020 [17]) using IDE RStudio. To fit RMA regression model we used lmodel2 package [18].

## Results

### The formula for the calibration of *k*

The formula is as follows:


$$ {K}_{\text c}(l)=k-\frac{s_{\text e}\sqrt{3m}}{l_{\max}-{l}_{\min}}\left(l-\frac{l_{\max}+{l}_{\min}}{2}\right) $$

where ${K}_{\text c}(l)$ is the thermal summation value after calibration with insect size, *k* is the thermal summation value (age at emergence) from the general thermal summation model, ${s}_{\text e}$ denotes standard error of *k*, $m$ is the number of temperatures used to derive *k*, ${l}_{\max}$ and ${l}_{\min}$ are maximal and minimal insect sizes (i.e. lengths in this paper) in a given species or population, and $l$ is an insect size (here length) for which we want to obtain ${K}_{\text c}$ (see Appendix for the second version of the formula, Supplementary Online Material).

### Derivation of the formula

The formula was derived based on the RMA regression model. The estimators of coefficients obtained by the RMA methods, for the linear model $y={\beta}_0x+{\beta}_1$, are as follows:

**Table:** 

	**Reduced major axis**
Loss function	$L\left(\boldsymbol{x},\boldsymbol{y}\right)=\sum_{i=1}^n\left({y}_i-{\hat{y}}_i\right)\left({x}_i-{\hat{x}}_i\right)$
Estimator of slope	${\hat{\beta}}_0=\mathit{\operatorname{sign}}\left(\sum_{i=1}^n\left({y}_i-\overline{\boldsymbol{y}}\right)\left({x}_i-\overline{\boldsymbol{x}}\right)\right)\sqrt{\frac{\sum_{i=1}^n{\left({y}_i-\overline{\boldsymbol{y}}\right)}^2}{\sum_{i=1}^n{\left({x}_i-\overline{\boldsymbol{x}}\right)}^2}}$
Estimator of intercept	${\hat{\beta}}_1=\overline{\boldsymbol{y}}-{\hat{\beta}}_0\overline{\boldsymbol{x}}$

Where $\overline{\boldsymbol{x}}=\frac{1}{n}\sum_{i=1}^n{x}_i$, $\overline{\boldsymbol{y}}=\frac{1}{n}\sum_{i=1}^n{y}_i$, ${\hat{x}}_i=\frac{y-{\hat{\beta}}_1}{{\hat{\beta}}_0}$, ${\hat{y}}_i={\hat{\beta}}_0x+{\hat{\beta}}_1$, $i=1,\dots, n$.

Estimators of the coefficients are constructed based on the estimators of two population parameters, i.e. a correlation and a standard deviation. Using the formulas: $sd\left(\boldsymbol{x}\right)=\sqrt{\frac{1}{n}\sum_{i=1}^n{\left({x}_i-\bar{\boldsymbol{x}}\right)}^2}$ and $cor\left(\boldsymbol{x},\boldsymbol{y}\right)=\frac{\frac{1}{n}\sum_{i=1}^n\left({y}_i-\overline{\boldsymbol{y}}\right)\left({x}_i-\overline{\boldsymbol{x}}\right)}{sd\left(\boldsymbol{x}\right) sd\left(\boldsymbol{y}\right)}$, the estimator of slope can be written as follows:


$$ {\hat{\beta}}_0=\mathit{\operatorname{sign}}\left( cor\left(\boldsymbol{x},\boldsymbol{y}\right)\right)\frac{sd\left(\boldsymbol{y}\right)}{sd\left(\boldsymbol{x}\right)} $$

Insect length ($l$) is our $x$-variable, and the “true *k*” (*k* for an individual insect evidence, hereafter ${k}_t$) is our $y$-variable. Recent papers suggest that $cor\left(\boldsymbol{l},{\boldsymbol{k}}_{\boldsymbol{t}}\right)<0$, so we will assume this [14, 15]. We will also assume that the “true *k*” is a random variable. The standard deviation of the “true *k*” can be estimated as $sd\left({\boldsymbol{k}}_{\boldsymbol{t}}\right)\approx \frac{s_e\sqrt{m}}{2}$, where ${s}_e$ is a standard error of *k* from the thermal summation model and $m$ is the number of temperatures used to derive the model (argumentation in the Appendix, Supplementary Online Material).

Since, the sample of insect lengths is a specific arithmetic sequence, its standard deviation can be approximated by the formula (argumentation in the Appendix, Supplementary Online Material):


$$ sd\left(\boldsymbol{l}\right)\approx \frac{\sqrt{3}\left({l}_{\max}-{l}_{\min}\right)}{6}. $$

Moreover, the mean of a sample of insect length can be approximated as follows:


$$ \overline{\boldsymbol{l}}\approx \frac{l_{\max}+{l}_{\min}}{2}. $$

The reasoning and assumptions outlined above provide the formula to estimate the line of RMA regression model and in effect the formula to calibrate *k* based on insect size:


$$ {K}_{\text c}^{\text{RMA}}(l)={K}_{\text c}(l)=k-\frac{s_{\text e}\sqrt{3m}}{l_{\max}-{l}_{\min}}\left(l-\frac{l_{\max}+{l}_{\min}}{2}\right). $$

### Validation of the formula

**Figure 1:**
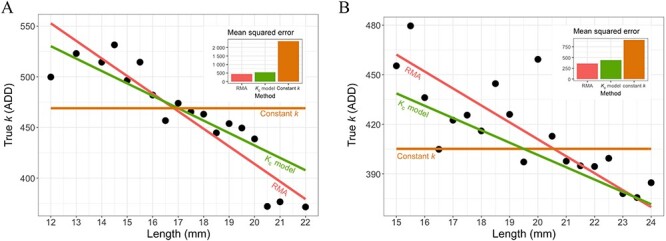
Comparison of the models: the reduced major axis (RMA) model—red line, the calibrated *k* (${K}_{\text c}$) model—green line, and the constant $k$ from the general model—orange line. Black points are size classes of the insects plotted against medians of the “true *k*”. (A) *Necrodes littoralis*; (B) *Creophilus maxillosus*.

In both species regression models (the RMA model for actual data and the ${K}_{\text c}$ model for the calibrations using the formula) revealed a much better representation of the “true *k*” than a constant *k* from the general thermal summation model. The MSE of the RMA and ${K}_{\text c}$ models were similar to each other, but much lower than the errors for the constant *k* (Figure 1).

**Figure 2:**
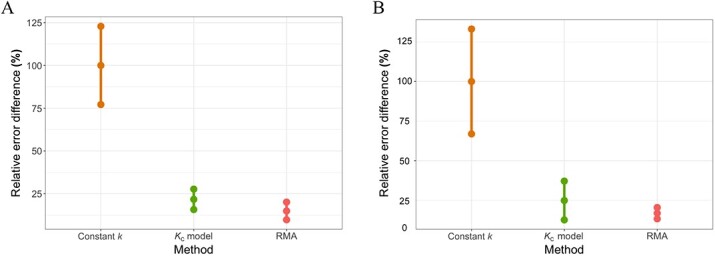
Comparison of the squared errors between the models calculated for unusually small and large insects only. The 100% reference point is a mean error of the constant *k*. Whiskers denote standard deviation of errors. (A) *Necrodes littoralis*; (B) *Creophilus maxillosus*. RMA: reduced major axis.

The comparison of the models calculated for unusually small and large insects only, revealed in both species the significant differences in the squared errors (ANOVA: *N. littoralis,*  $F=7,P=0.00068$; *C. maxillosus*, $F=3.392,P=0.0253$, Figure 2). The errors for ${K}_{\text c}$ and RMA models were similar and much smaller than for constant *k* (by ~80% in the case of *N. littoralis* and by ~75% in the case of *C. maxillosus*, Least Significant Difference *post hoc* test with Benjamini & Hochberg *P*-value adjustment, Figure 2).

## Discussion

The formula developed in this article, enables calibration of the thermal summation value needed for insect emergence (i.e. *k*) based on simple measurements of adult insects reared from larvae or pupae sampled on a death scene. Most importantly, this calibration does not require the “*k versus* size” model that describes the relationship between insect size at maturity and *k* for the species. Therefore, the formula facilitates calibration of *k*, since it is not necessary to collect data needed to derive the “*k versus* size” model. Previously, the calibration of *k* was only possible by using the “*k versus* size” model [14, 15]. This is the main advantage of the approach presented in this article.

The formula was validated using *N. littoralis* and *C. maxillosus* development data, with highly satisfying results. The calibration was particularly useful while analyzing unusually small and large insects. In such cases (for this example the benefits were calculated for three smallest and three largest beetles in each species) it reduced the inaccuracy of *k* from the general model on average by ~25 accumulated degree-days (ADD) in the case of *C. maxillosus* (~3 days under constant 20°C and D_0_ = 11.7°C) and ~40 ADD in the case of *N. littoralis* (~3.5 days under constant 20°C and D_0_ = 8.5°C). When insect size was close to the average for the population, the calibration provided no substantial improvement over *k* from the general thermal summation model. However, for the sake of standardization and simplicity, we postulate to use the calibration protocol in all cases.

The main advantage of the formula is that it may be used without the “*k versus* size” model. Accordingly, it may provide calibrations of *k* for species, in case of which the relationship between *k* and insect size was not analyzed. To facilitate this kind of use we retrieved from the literature the necessary size data (Table 1) and calculated the detailed calibration formulas for selected insect species of forensic significance (Table 2).

**Table 1:** Size ranges of adult insects (body length) referred by different sources for selected species of forensically important insects that breed in large carcasses in Central Europe [26–30].

	**Size range according to different sources (mm)**			
**Species**	**Source I**	**Source II**	**Source III**	**Source IV (Wikipedia)** ^a^
*Chrysomya albiceps* (Diptera: Calliphoridae)	$5-12$ [31]			$6-9$
*Lucilia sericata* (Diptera: Calliphoridae)	$5-12$ [31]			$10-14$
*Phormia regina* (Diptera: Calliphoridae)	$6-10$ [31]			
*Fannia canicularis* (Diptera: Fanniidae)	$3.5-7$ [32]	$4.5-7$ [33]		$3.5-6$
*Necrobia rufipes* (Coleoptera: Cleridae)	$3.5-4.5$ [34]	$4-5.5$ [35]	$3.5-7$ [36]	$3.5-7$
*Omosita colon* (Coleoptera: Nitidulidae)	$2-3.6$ [37]	$2-3.6$ [38]		$2-3.5$
*Necrodes littoralis* (Coleoptera: Silphidae)	$15-25$ [39]	$16-25$ [40]	$12-22$ [15]	$15-25$
*Thanatophilus sinuatus* (Coleoptera: Silphidae)	$9-12$ [39]	$9-12$ [40]		$9-12$
*Thanatophilus rugosus* (Coleoptera: Silphidae)	$8-12$ [39]	$10-13$ [40]		$10-14$
*Creophilus maxillosus* (Coleoptera: Staphylinidae)	$15-25$ [41]	$15-23$ [42]	$15-24$ [14]	$12-18$
*Nasonia vitripennis* (Hymenoptera: Pteromalidae)	$1.3-2.2$ [43]			$2-3$

a The status at 14 November 2021, English or German version was used.

**Table 2:** Detailed formulas and data necessary for their derivation for selected species of forensically important insects that breed in large carcasses in Central Europe [26–30].

**Species**	**General *k* (SE) (ADD/ADH)**	**Size range used (mm)**	${\boldsymbol{K}}_{{\text c}}$ **– a detailed formula**
*C. albiceps*	$143.52$ ADD $(5.61)$ [21]	$5-12$ [31]	${K}_{\text c}(l)=174.74-3.67l$
*L. sericata*	$6 \, 023.2$ ADH $(297.2)$ [44]	$5-12$ [31]	${K}_{\text c}(l)=7\,676.99-194.56l$
*P. regina*	$281$ ADD $(3.6)$ [12]	$6-10$ [31]	${K}_{\text c}(l)=308.89-3.49l$
*F. canicularis*	$481.73$ ADD $(9.89)$ [45]	$3.5-7$ [32]	${K}_{\text c}(l)=554.41-13.84l$
*N. rufipes*	$591$ ADD $(39.53)$ [46]	$3.5-7$ [36]	${K}_{\text c}(l)=842.57-47.92l$
*O. colon*	$514.1$ ADD $(8.7)$ [47]	$2-3.6$ [38]	${K}_{\text c}(l)=578.69-23.07l$
*N. littoralis*	$469.89$ ADD $(24.59)$ [15]	$15-25$ [39]	${K}_{\text c}(l)=710.82-12.05l$
*T. sinuatus*	$360.46$ ADD $(10.75)$ [7]	$9-12$ [39]	${K}_{\text c}(l)=532.88-16.42l$
*T. rugosus*	$362.758$ ADD $(4.97)$ [20]	$8-12$ [39]	${K}_{\text c}(l)=415.47-5.27l$
*C. maxillosus*	$405.156$ ADD $(14.63)$ [13]	$15-25$ [41]	${K}_{\text c}(l)=548.5-7.17l$
*N. vitripennis*	$4\,768.8$ ADH $($431.5$)$ [48]	$1.3-2.2$ [43]	${K}_{\text c}(l)=8\,328.49+2034.1l$

a ADD: accumulated degree-days; ADH: accumulated degree-hours; SE: standard error.

The main weakness of the formula is that it was derived theoretically, so it should be used with caution. Moreover, the formula was tested only for the laboratory populations of two beetle species originating from a single geographical area. Therefore, its generalization beyond these species and populations without further tests could be risky.

Age and size of insects at maturity were found to be usually negatively correlated in phytophagous and predatory insects, and usually positively correlated in parasitoid insects [19]. Based on these general patterns, we assumed that among carrion insects necrophagous or predatory species reveal negative correlation between *k* and size and parasitoid species reveal positive correlation. This assumption was supported by the only two datasets for carrion insects (a necrophagous *N. littoralis* and a predatory *C. maxillosus*), in case of which *k* and size were negatively correlated [14, 15]. Therefore, in our Table 2 in all the species, apart from *Nasonia vitripennis* that is a parasitoid species, we presumed a “–” correlation sign. However, we encourage researchers in forensic entomology to analyze the correlation between *k* and size in other carrion insects and to further test the above assumption.

A review of size ranges for selected species of forensically-important insects revealed substantial differences between the reference sources (Table 1). To some extent, these differences may reflect natural differences between geographic populations that were used to make these measurements. They may be also attributed to the differences in measurement protocols. This is also possible that the differences result from poor representativeness of certain samples, which were used to make the measurements. Therefore, while calibrating *k* for the other species or using the other datasets, one should employ size range for the closest geographic population and measure size of insect evidence using the same measurement protocol, which was used to provide the size data. Moreover, when various ranges are available for the same or close populations, the broadest range should be used, since it represents natural variation of size in the reference population with probably the highest accuracy.

Forensic entomologists usually use general data that describes average values or trends for the large groups of objects, e.g. *k* from the general thermal summation model is such an average value for the large insect group. However, pieces of insect evidence are specific objects and we should analyze them using more specific data, since it will increase the accuracy of the minimum PMI estimation. As a rule, forensic entomologists use developmental data for species or their geographic subpopulations [13, 20, 21]. More detailed data and tools to collect them were developed only recently, with the focus on sex and size of insects [8–10, 14, 15, 22–25]. Further studies in this field are necessary and in particular we should look for the new traits and tools that could be useful to calibrate developmental data for narrow subpopulations of insect species. While our calibration formula allows the use of developmental data for particular size groups of insects, therefore the data that are highly detailed, we believe that similar calibration protocols could yield even more detailed data. For instance, supplementing the formula with correction factors for females and males could provide the data for particular size groups of separately female and male insects. More studies are however necessary, to elucidate general patterns for the sex-related differences in *k*.
